# Effect of body mass index on clinical outcome and all-cause mortality in patients undergoing transcatheter aortic valve implantation

**DOI:** 10.1007/s12471-017-1003-2

**Published:** 2017-05-23

**Authors:** M. Abawi, R. Rozemeijer, P. Agostoni, R. C. van Jaarsveld, C. S. van Dongen, M. Voskuil, A. O. Kraaijeveld, P. A. F. M. Doevendans, P. R. Stella

**Affiliations:** 1 Department of Cardiology, University Medical Center Utrecht, Utrecht, The Netherlands; 2 Department of Cardiology, St. Antonius Hospital, Nieuwegein, The Netherlands

**Keywords:** Transcatheter aortic valve implantation, TAVR, Body mass index, Obesity, Overweight, Obesity paradox

## Abstract

**Objectives:**

To assess the effect of body mass index (BMI) on outcome among patients with severe aortic stenosis (AS) admitted for transcatheter aortic valve implantation (TAVI).

**Background:**

Being overweight or obese is associated with improved outcome following certain medical treatments, suggesting the existence of a BMI paradox. However, the relationship between BMI and mortality after TAVI remains controversial.

**Methods:**

Patients were classified according to World Health Organisation criteria such as normal weight, overweight, or obesity according to their BMI (18.5 to 24.9 kg/m^2^, 25.0 to 29.9 kg/m^2^, and ≥30.0 kg/m^2^, respectively).

**Results:**

A total of 549 consecutive patients (age: 80.2 ± 7.5 years; logistic European system for cardiac operative risk evaluation [EuroSCORE]: 17.3 ± 9.9%) who underwent TAVI for AS were included. Of these patients, 43% (*n* = 237) had normal weight, 36% (*n* = 200) were overweight, and 20% (*n* = 112) were obese. There were no differences in peri-operative bleeding or vascular complication rates between the groups. All-cause mortality after 30 days, and 1 year, were higher in normal weight patients compared with overweight and obese patients (7% vs. 5 and 4%, *p* = 0.383, and 19% vs. 9 and 10%, *p* = 0.006, respectively). After adjustment for several confounding factors, overweight was associated with a decreased 30-day and 1‑year all-cause mortality outcome (hazard ratio [HR] 0.69; 95% confidence interval [CI] 0.47–0.99, and HR 0.65; 95% CI 0.45–0.94, respectively).

**Conclusions:**

Despite the well-documented adverse effects of increased body weight on health, being overweight is associated with improved survival following TAVI when compared with normal weight.

## Introduction

Overweight and obesity are the fifth leading modifiable cause of death in the world, accounting nearly 3.4 million deaths annually [1]. The prevalence of obesity, often defined as body mass index (BMI), has been increasing dramatically [1]. According to available data, more than two-thirds of adults in the United States, and more than 2.1 billion people worldwide, suffer from obesity [1, 2]. Although it has been suggested that obesity occurs because of an energy imbalance between caloric intake and expenditure, the resulting energy excess and associated weight gain reflects a complex interaction between genes, epigenetic markers, environment, and lifestyle [2–4].

According to the Framingham Heart study, conducted among participants (age: 30–49 years) with no cardiovascular disease at baseline, overweight and obesity were associated with a decrease in life expectancy and increased early mortality during the follow-up period of ≥4 years [5]. Accordingly, another population-based cohort reported an increased risk of all-cause mortality among elderly (≥85 years) obese participants [6]. In addition to the risk of mortality, obesity is an underlying promotor of systemic metabolic dysfunction, i. e., dyslipidaemia, decreased insulin sensitivity, hyperinsulinaemia, hyperglycaemia, and hypertension [2, 4].

However, despite the well-documented adverse effects of overweight or obesity on general health status, being overweight or obese is associated with better survival in patients undergoing medical interventions [7], vascular surgery [8], cardiovascular intervention [9], and in patients who are hospitalised for acute decompensated heart failure [10, 11]. These observations led to the concept of reverse epidemiology, also known as the obesity paradox. The obesity paradox states that a higher BMI may, counter-intuitively, be linked to improved survival in certain patient groups. However, these observations do not support common practice where weight loss is recommended prior to cardiac treatments.

Contradicting data exist regarding the effect of BMI on outcome in patients with aortic stenosis (AS). In one study (*n* = 1664) overweight and obese patients with AS were at increased risk for mortality, whereas another study (*n* = 400) found contradictory results [12, 13]. Although there are few data regarding the effect of BMI on outcome among patients with AS who undergo transcatheter aortic valve implantation (TAVI), several cohort studies showed contradicting or supporting results [14–20]. For instance, a large French Aortic National CoreValve and Edwards (FRANCE-2) registry showed improved survival outcome among overweight and obese individuals undergoing TAVI [14]. Overweight or obesity was associated with improved survival following TAVI in other cohort studies as well [21]. However, a recent study did not find such a paradoxical relationship [19]. Therefore, the current study aimed to assess the effects of body mass index on short and long-term all-cause mortality in patients undergoing TAVI in the current era.

## Methods

We performed a retrospective observational cohort study encompassing all eligible consecutive patients who underwent TAVI between September 2008 and October 2016 at the University Medical Center Utrecht, Utrecht, the Netherlands. All demographic and peri-procedural data were prospectively collected in our dedicated database and retrospectively analysed in this study. All patients gave informed consent for the procedure and due to the retrospective nature of the study design, ethics committee approval was waived.

### Body mass index

BMI was defined as the weight in kilograms divided by the square of the height in meters. The weight and height of all patients were prospectively collected at hospital admission before the TAVI procedure. Baseline and clinical data were stratified by BMI categories according to the World Health Organisation (WHO) criteria as normal weight, overweight, and obese (18.5 to 24.9 kg/m^2^, 25.0 to 29.9 kg/m^2^, and ≥30.0, respectively).

### TAVI procedure

All patients had been judged inoperable or at high operative risk by the Heart Team and required consensus of at least one interventional cardiologist and one cardiac surgeon. Motivations to refuse surgical aortic valve replacement (SAVR) in patients were: 1) logistic European system for cardiac operative risk evaluation [EuroSCORE] ≥15%, or 2) the presence of contra-indications to cardiac surgery, e. g. porcelain aorta, frailty or patent grafts in proximity of the sternum. Frailty was subjectively measured prior to allocating TAVI by an interventional cardiologist and/or cardiothoracic surgeon based on the informal ‘eyeball test’ (including cognition function, physical weakness and walk speed). Access site was evaluated based on the measurements of pre-procedural multislice computed tomography scan. Valve implantation was performed either via the transfemoral or non-transfemoral approach (transapical or direct aortic). General anaesthesia or conscious sedation was used according to current local practice.

### Study endpoints

Main endpoint of this study was all-cause mortality at 30 days and 1 year after TAVI. All clinical outcomes were documented during the hospital stay, in compliance with the Valve Academic Research Consortorium-2 (VARC‑2) criteria and compared across all 3 BMI categories [22]. Vascular complications were documented for all procedural ‘access sites’, defined as any location traversed by a guide-wire, a catheter or a sheath during the procedure, including arteries, veins, left ventricular apex and the aorta. For the evaluation of postoperative delirium (POD) by the nurse or attending physician, a Delirium Observational Screening (DOS) scale score was rated at the end of every shift, according to the local protocol [23].

### Statistical analysis

Categorical variables were expressed as frequencies and percentages and compared with the One-way ANOVA, Chi-squared test or Fisher’s exact test, when appropriate. We applied Bonferroni’s correction in case of multiple comparisons. Continuous variables were expressed as mean and standard deviation if normally distributed or as median [interquartile range] if skewed and compared with the Student’s t‑test or the Mann-Whitney *U* test, respectively.

The association between BMI as a categorical variable and all-cause mortality was analysed using Kaplan–Meier survival estimates and the Log-Rank test. We developed a Cox regression model with selected variables with a *p*‑value < 0.10 to isolate the association of BMI with all-cause mortality. All statistical analyses were carried out using the IBM Statistical Package for Social Science for Windows, version 24.0 (IBM Corp., Armonk, New York, USA).

## Results

### Patient characteristics

We included a total of 562 consecutive patients who underwent TAVI for severe AS in the study. Because of the small sample size, we excluded patients (*n* = 13) with BMI ≤ 18.5 kg/m^2^, leaving 549 (98%) patients for the final analysis. Patient characteristics of all patients included in this study are given in Table 1.

**Table 1:** Baseline characteristics stratified according to the BMI categories

	All patients (*n* = 549) (*n* (%))	NW (*n* = 237)	OW (*n* = 200)	O (*n* = 112)	*p*-value, overall	NW vs. OW	NW vs. O	OW vs. O
Age, years	80.2 ± 7.5	80.8 ± 7.5	80.5 ± 7.3	78.4 ± 7.5	0.004	1.000	0.003	0.025
Gender, male	241 (44)	113 (48)	96 (48)	32 (29)	0.001	1.000	0.002	0.003
BMI, kg/m^2^	26.6 ± 4.4	22.8 ± 1.5	27.2 ± 1.4	33.3 ± 2.8	0.000	0.000	0.000	0.000
Logistic EuroSCORE	17.3 ± 9.9	18.1 ± 10.6	16.5 ± 8.5	17.4 ± 10.5	0.417	0.637	1.000	1.000
Frailty	184 (34)	80 (34)	60 (30)	44 (39)	0.248	1.000	0.922	0.289
NYHA class ≥ III	305 (58)	131 (58)	100 (52)	74 (67)	0.041	0.740	0.313	0.035
Estimated GFR, ml/min	56.3 ± 22.5	56.4 ± 23.5	57.6 ± 20.3	53.8 ± 24.5	0.460	1.000	0.867	0.702
Porcelain aorta	58 (11)	25 (11)	24 (12)	9 (8)	0.551	1.000	1.000	0.828
Diabetes mellitus	175 (32)	54 (23)	62 (31)	59 (53)	0.000	0.180	0.000	0.000
Hypertension	329 (60)	129 (54)	120 (60)	80 (71)	0.010	0.703	0.007	0.142
Dyslipidemia	185 (34)	63 (27)	77 (39)	45 (40)	0.008	0.025	0.036	1.000
Smoking (current/prior)	178 (32)	81 (34)	67 (34)	30 (27)	0.356	1.000	0.508	0.675
Coronary artery disease	261 (48)	114 (48)	90 (45)	57 (51)	0.591	1.000	1.000	0.956
Prior myocardial infarction	103 (19)	42 (18)	37 (19)	24 (21)	0.705	1.000	1.000	1.000
Prior PCI	200 (36)	93 (39)	71 (36)	36 (32)	0.412	1.000	0.598	1.000
Prior CABG	90 (16)	33 (14)	36 (18)	21 (19)	0.390	0.758	0.770	1.000
Peripheral artery disease	118 (22)	59 (25)	36 (18)	23 (21)	0.209	0.243	1.000	1.000
Atrial fibrillation	174 (32)	78 (33)	58 (29)	38 (34)	0.580	1.000	1.000	1.000
Active malignancy	60 (11)	27 (11)	25 (13)	8 (7)	0.331	1.000	0.707	0.440
COPD	109 (20)	50 (21)	37 (19)	22 (20)	0.793	1.000	1.000	1.000
Pulmonary hypertension	38 (7)	16 (7)	12 (6)	10 (9)	0.620	1.000	1.000	1.000
Prior TIA or stroke	108 (20)	47 (20)	41 (21)	20 (18)	0.850	1.000	1.000	1.000
*Medication use*								
CCBs	120 (22)	56 (24)	40 (20)	24 (21)	0.653	1.000	1.000	1.000
Beta blockers	305 (56)	128 (54)	107 (54)	70 (63)	0.252	1.000	0.410	0.377
Antiarrhythmics	40 (7)	16 (7)	12 (6)	12 (11)	0.281	1.000	0.553	0.375
Diuretics	345 (63)	137 (58)	128 (64)	80 (71)	0.044	0.543	0.042	0.576
ARBs	99 (18)	36 (15)	31 (16)	32 (29)	0.005	1.000	0.007	0.012
Aspirin	298 (54)	135 (57)	102 (51)	61 (55)	0.459	0.641	1.000	1.000
Lipid-lowering agents	310 (57)	121 (51)	116 (58)	73 (65)	0.039	0.432	0.039	0.657
Insulin	70 (13)	16 (7)	29 (15)	25 (22)	0.000	0.044	0.000	0.134
*Echocardiography data*								
LVEF	54.6 ± 17.8	53.1 ± 16.6	52.6 ± 16.7	61.3 ± 20.2	0.000	1.000	0.001	0.000
LVEF ≤ 30	46 (9)	19 (9)	22 (12)	5 (5)	0.171	0.948	0.838	0.188
Peak aortic gradient, mm Hg	66.0 ± 23.3	66.3 ± 24.0	65.3 ± 23.5	66.4 ± 21.5	0.742	1.000	1.000	1.000
Mean aortic gradient, mm Hg	40.0 ± 17.1	40.3 ± 17.6	39.8 ± 16.5	39.7 ± 16.5	0.962	1.000	1.000	1.000
*Procedural*								
General anaesthesia	154 (28)	72 (30)	53 (27)	29 (26)	0.567	1.000	1.000	1.000
Non-transfemoral^a^	91 (17)	46 (19)	32 (16)	13 (12)	0.181	1.000	0.230	0.951
PPR, ≥mild	32 (6)	21 (9)	6 (3)	5 (5)	0.025	0.025	0.310	1.000

*NW* normal weight (18.5 ≤ BMI ≤ 24.9), *OW* overweight (25.0 ≤ BMI ≤ 29.9), *O* obese (BMI ≥ 30.0), *BMI* body mass index, *GFR* glomerular filtration rate, *PCI* percutaneous coronary intervention, *CABG* coronary artery bypass grafting, *COPD* chronic obstructive pulmonary disease, *TIA* transient ischaemic attack,* CCBs* calcium channel blockers, *ARB’s* angiotensin II receptor blockers, *LVEF* left ventricular ejection fraction, *PPR* peri-prosthetic aortic valve regurgitation

^a^Transapical/direct aorta

**Fig. 1:**
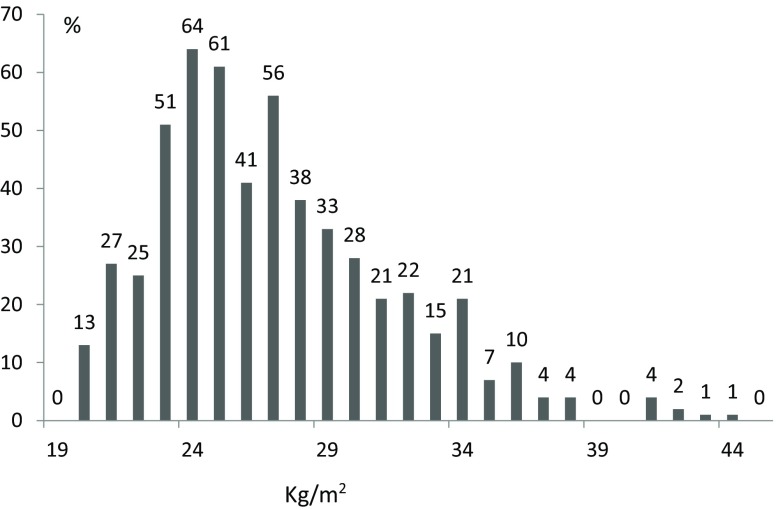
Distribution of body mass index

According to the BMI categories, 43% (*n* = 237) had normal weight, 36% (*n* = 200) were overweight, and 20% (*n* = 112) were obese. BMI distribution is graphically presented in Fig. 1. Obese patients were relatively younger than normal weight and overweight patients (78.4 ± 7.5 vs. 80.8 ± 7.5 and 80.5 ± 7.3, *p* = 0.004, respectively); were more often female (71% vs. 52 and 52%, *p* = 0.001, respectively); obese patients had, non-surprisingly, a higher prevalence of diabetes mellitus (53% vs. 23 and 31%, *p* ≤ 0.001, respectively), hypertension (71% vs. 54 and 60%, *p* = 0.010, respectively), hypercholesterolaemia (40% vs. 27 and 39%, *p* = 0.008, respectively). Obese patients had a higher left ventricular ejection fraction (LVEF) compared with normal weight and overweight patients (61.3 ± 20.2 vs. 53.1 ± 16.6, and 52.6 ± 16.7%, *p* ≤ 0.001, respectively). After the procedure, normal weight was associated with mild/or more than mild periprosthetic aortic valve regurgitation (PPR). No differences were observed in procedural features between the groups.

### Clinical outcomes

In-hospital outcomes are summarised in Table 2. Median follow-up time was 682 [interquartile range: 328–1270] days, and 33% (*n* = 181) deaths occurred during the follow-up period, with the highest rate among normal weight patients compared with overweight and obese patients (38.4% vs. 27.5% and 31.3%, *p* = 0.049, respectively). At 30-day follow-up, as well as 1‑year follow-up, all-cause mortality rates were higher in normal weight patients compared with overweight and obese patients (6.8% vs. 4.5%, and 3.6%, *p* = 0.386; and 18.6% vs. 9.0%, and 9.8%, *p* = 0.006, respectively). There were no differences in in-hospital bleeding or vascular complications between the groups.

**Table 2:** Clinical outcomes

	All patients (*n* (%))	NW	OW	O	*p*-value, overall	NW vs. OW	NW vs. O	OW vs. O
*Bleeding complications*								
Life-threatening or major	78 (14)	35 (15)	27 (14)	16 (14)	0.931	1.000	1.000	1.000
Minor	82 (15)	31 (13)	35 (18)	16 (14)	0.424	0.593	1.000	1.000
*Vascular complications*								
Major	76 (14)	33 (14)	27 (14)	16 (14)	0.980	1.000	1.000	1.000
Minor	74 (14)	29 (12)	30 (15)	15 (13)	0.701	1.000	1.000	1.000
AKI stage ≥ 2	29 (5)	18 (8)	7 (4)	4 (4)	0.108	0.170	0.350	1.000
PPI	54 (10)	24 (10)	15 (8)	15 (13)	0.246	1.000	1.000	0.292
New onset AF	72 (13)	34 (14)	27 (14)	11 (10)	0.495	1.000	0.731	1.000
TIA or stroke	22 (4)	8 (3)	8 (4)	6 (5)	0.392	1.000	1.000	1.000
POD	77 (14)	45 (19)	19 (10)	13 (12)	0.012	0.013	0.189	1.000
Infection^*a*^	38 (7)	19 (8)	13 (7)	6 (5)	0.631	1.000	1.000	1.000
In-hospital stay, days	6.7 ± 5.4	7.3 ± 6.3	6.1 ± 20	6.7 ± 5.8	0.057	0.050	1.000	0.864
In-hospital mortality	22 (4)	12 (5)	6 (3)	4 (4)	0.395	0.823	1.000	1.000

*NW* normal weight (18.5 ≤ BMI ≤ 24.9), *OW* overweight (25.0 ≤ BMI ≤ 29.9), *O* obese (BMI ≥ 30.0), *AKI* acute kidney injury, *PPI* permanent pacemaker implantation, *AF* atrial fibrillation, *TIA* transient ischaemic attack, *POD* postoperative delirium

^a^Infections (Urinary tract, OR access site, OR pneumonia, OR combined).

**Fig. 2:**
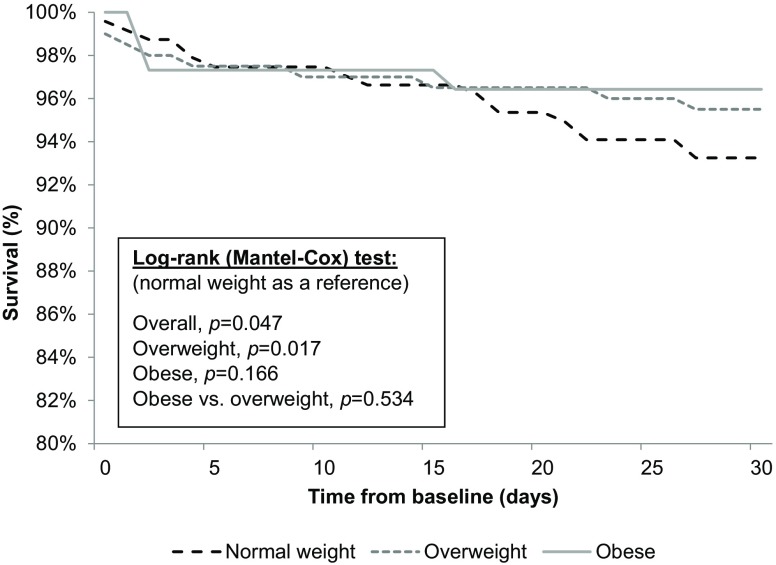
Thirty-day all-cause mortality, graphically presented by Kaplan–Meier survival curves

**Fig. 3:**
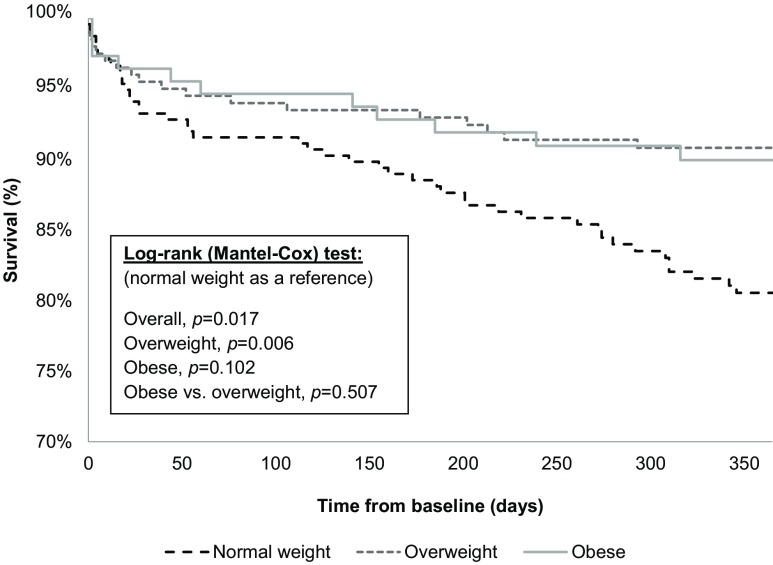
One-year all-cause mortality, graphically presented by Kaplan–Meier survival curves

Unadjusted survival is presented as a Kaplan–Meier curve in Figs. 2 and 3. Estimated survival rates varied significantly among the groups after 30 days (*p* = 0.047, log-rank test) and 1 year (*p* = 0.017, log-rank test). Patients with normal weight with BMI 18.5–24.9 kg/m^2^ had the highest mortality risk, whereas overweight patients had the lowest mortality risk.

Univariate and multivariate analysis results of the association between BMI and 30-day and 1‑year mortality are shown in Table 3. After adjustment for baseline and peri-procedural covariates, i. e., age, gender, New York Heart Association class ≥ III, diabetes mellitus, hypertension, dyslipidaemia, left ventricular ejection fraction, calcium channel blockers, beta blockers, antiarrhythmics, diuretics, angiotensin II receptor antagonists, aspirin, lipid-lowering agents, insulin, PPR ≥ mild, postoperative delirium, and hospital stay, only overweight was associated with a decreased 30-day and 1‑year all-cause mortality rate compared with normal weight and obesity (overweight adjusted model at 30 days: hazard ratio [HR] 0.69; 95% confidence interval [CI] 0.47–0.99; adjusted model at 1 year: HR 0.65; 95% CI 0.45–0.94, respectively). However, there was no association between BMI as a continuous variable and mortality.

**Table 3:** Effect of body weight on all-cause mortality during follow-up

	Univariate HR (95% CI)	*p*	Multivariate^a^ HR (95% CI)	*p*
*At 30-day follow-up*				
Body mass index^b^	0.98 (0.95–1.01)	0.258	0.98 (0.94–1.02)	0.374
Normal weight vs. overweight	0.70 (0.50–0.98)	0.038	0.68 (0.45–0.95)	0.027
Normal weight vs. obese	0.79 (0.54–1.17)	0.237	0.81 (0.50–1.29)	0.370
Overweight vs. obese	1.13 (0.74–1.72)	0.583	1.18 (0.71–1.96)	0.520
*At 1‑year follow-up*				
Body mass index^b^	0.98 (0.94–1.01)	0.189	0.97 (0.94–1.02)	0.217
Normal weight vs. overweight	0.65 (0.47–0.91)	0.013	0.69 (0.43–0.88)	0.009
Normal weight vs. obese	0.74 (0.50–1.10)	0.136	0.70 (0.43–1.12)	0.133
Overweight vs. obese	1.14 (0.75–1.74)	0.547	1.19 (0.72–1.96)	0.497

*HR* hazard ratio, *CI* confidence interval

^a^Adjusted for: age, gender, New York Heart Association (NYHA) class ≥ III, diabetes mellitus, hypertension, dyslipidaemia, left ventricular ejection fraction (LVEF), calcium channel blockers, beta blockers, antiarrhythmics, diuretics, angiotensin II receptor blockers, aspirin, lipid-lowering agents, insulin, periprosthetic aortic valve regurgitation (PPR) ≥mild, postoperative delirium, hospital stay

^b^As a continuous variable

## Discussion

In the present study, we aimed to evaluate the impact of body mass index on all-cause mortality and clinical outcome in patients undergoing TAVI. After adjustment, being overweight was associated with decreased 30-day and 1‑year all-cause mortality, while there was no association found between obesity and mortality outcomes following TAVI. Furthermore, there were no differences observed in postoperative bleeding or vascular complications between the BMI categories.

Considering the aging population, the global prevalence of overweight and obesity is expected to rise [12]. AS is the predominant type of valvular heart disease among elderly and associated with poor prognosis [24]. Prevalence of AS is 3%, increasing with age up to 10% in adults ≥80 years [25]. Currently, TAVI has emerged as a valuable option to treat severe AS in elderly patients considered to be inoperable or at high surgical risk for SAVR [26]. However, fewer data exist regarding body weight management in patients undergoing TAVI.

Interestingly, we observed decreased short-term and long-term mortality outcomes after TAVI among overweight patients. According to our knowledge, this is the first time such a short-term effect of overweight on mortality outcomes after TAVI has been shown. Our findings are in line with literature findings including patients who were admitted for first-time coronary artery bypass (CABG) or combined CABG/aortic valve replacement surgery, patients after coronary angiography for diagnosis of acute coronary syndrome, and among patients undergoing cardiac surgery [27–29]. While some studies reported no effect of being overweight on mortality outcome after TAVI [15, 19], others reported long-term positive effect of overweight on mortality outcome after TAVI [14, 17, 18]. Although current guidelines are advising weight loss and prevention of overweight and obesity, these counter-intuitive findings regarding the positive association between overweight/obesity and mortality may create the impression that an intentional weight loss may not always be favourable.

Generally, obesity has been associated with adverse clinical health status [4], however, we observed no association between obesity and mortality after TAVI. Our results are in line with some previous reports [15, 18, 19], however, several other studies including a meta-analysis, found a beneficial effect of obesity on mortality outcome after TAVI [14, 17, 21]. In addition, inconstancies in these contradictive observations could be explained by unhealthy metabolic profile (i. e., hypertension, dyslipidaemia, diabetes) of obese individuals included in our study that may have influenced the results [30].

Although we observed no association between BMI as a continuous variable and mortality, several other studies reported a gradual reduction in death rate for every increment in BMI unit (kg/m^2^) during short-term or long-term follow-up after TAVI [15–17]. However, a recent study among patients (*n* = 4571) undergoing TAVI demonstrated that an increase in BMI was associated with higher risk of mortality in patients with elevated BMI (>32 kg/m^2^) [20]. Moreover, a ‘U’ shape association between BMI a continuous variable and mortality was found among patients with diabetes, acute heart failure and in patients undergoing cardiac surgery [29, 31, 32].

While performing TAVI in overweight or obese individuals may be challenging due to vascular access site and fluoroscopic visualisation [33], there were no differences in vascular and bleeding complications observed between the BMI groups in our cohort. These findings are in line with a recent meta-analysis evaluating the effect of BMI on outcome after TAVI [21]. This could be explained by improved TAVI technique and sustained efficacy of TAVI. However, previous studies using early-generation transcatheter aortic valves and techniques reported higher postoperative complication rates among overweight/obese and underweight individuals after TAVI. For instance, in a multi-centre study (*n* = 940), higher rates of postoperative minor stroke, minor vascular complications and acute kidney injury stage 1 were observed among obese individuals following TAVI [15]. Consistent with these findings, another study (*n* = 409) reported higher incidence of major postoperative vascular complications and a trend toward more major and life-threatening bleeding events among obese patients after TAVI [17]. Furthermore, a higher rate of major vascular complications was observed in patients with underweight. However, according to another study, BMI < 20 compared with BMI > 20 was not associated with adverse events following TAVI [34]. Inconsistencies in reported results could be explained by different definitions of BMI, i. e., BMI either as a categorical or continuous variable, which could lead to uncertainty in defining the cut-off points and interpretation of results. Therefore, studies should report their results according to the standardised BMI classification, i. e., WHO classification.

The mechanism behind the obesity paradox remains unclarified [35]. However, there are several possible factors that could explain the paradoxical effect of BMI on clinical outcomes. For instance, excess body weight may increase metabolic reserve and counteract the negative effects of acute injuries. Furthermore, patients with ischaemic heart failure appear to have a higher level of TNF-a concentrations compared with those with a non-ischaemic aetiology [36]. Moreover, adipose tissue has been shown to produce TNF-a receptors [37], therefore, overweight and obese patients may have a protective buffer from the negative effect of increasing TNF-a by producing higher levels of these receptors. Furthermore, several other investigators argue that the obese group, consisting of younger individuals, seeks medical care earlier, is treated medically more aggressively, and therefore benefits more from medical and interventional treatments [38]. However, these differences did not affect the outcome after adjustments in the multivariate model, even though obese individuals in our cohort were younger and used more baseline medications, i. e., diuretics, angiotensin II receptor antagonists, lipid-lowering agents, or insulin, compared with the normal weight group.

BMI either as a continuous or categorical variable has been frequently used to define body weight. However, BMI does not discriminate between the component of body fat, the type and location of fat in the body, or the degree of metabolic diseases that it can cause. In the clinical setting, high muscle mass/low fat has been associated with improved survival in patients with cardiovascular disease [39]. Accordingly, loss of muscle mass has been associated with increased mortality in patients undergoing TAVI [40]. That is why future studies are necessary to determine the most favourable body weight to improve outcome after TAVI.

This study has several important limitations. First, this retrospective, single-centre analysis is subjected to the limitations common to this type of analysis. An observational analysis, including the current study, cannot prove or disprove the existence of a paradoxical relationship between BMI and mortality. Second, we used BMI as a surrogate of body weight. However, combining BMI and measures of central obesity, such as waist circumference and waist-to-hip ratio, may be more valuable in the assessment of mortality risk after TAVI, since central obesity predicts mortality more reliably than BMI alone in patients with coronary heart disease [41]. Third, in contrast to other findings, only overweight was associated with decreased mortality in patients in our cohort. However, it is possible that patients with a more severe profile of comorbidities and a high surgical risk for TAVI were refused to undergo TAVI, which could have affected current results. Fourth, we may not have included all possible (unknown) confounding factors that may have influenced the results. For instance, to address the likely bias attributed to patients with cachexia, we excluded patients with BMI < 18.5. However, as most elderly suffer from lower muscle mass, this will introduce bias attributable to unassured confounders [40]. Fifth, we could not address the effect of BMI changes over time, which may have influenced our results. Finally, although late mortality after TAVI may be attributed to the non-cardiac causes [42], we could not address the cause of death in patients included in this study. Future studies are needed to evaluate cause of death to provide a better understanding of the mechanism of the observed association between BMI and mortality.

## Conclusions

Being overweight is associated with improved survival after TAVI. Furthermore, TAVI is safe in different BMI groups with respect to the postoperative complications rate.
